# Supplementary material for: Algorithm, expert, or both? Evaluating the role of feature selection methods on user preferences and reliance
Source: PLoS One. 2025 Mar 7;20(3):e0318874. doi: 10.1371/journal.pone.0318874 (PMC11888136; doi:10.1371/journal.pone.0318874)
Supplement: S1 Text — (PDF) [file pone.0318874.s001.pdf]

S1 Text

"Dear Participant, Thank you for your interest in our study. This page will provide you with a detailed set of instructions to guide you through our study. Please read this carefully before starting.

Study Overview In this study, you aim to make correct decisions in 40 classification tasks in two domains. In each task, you will be presented with 12 pieces of information to decide. The more correct decisions you make, the higher your bonus payment will be.

Artificial Intelligence During this task, you will be supported by an Artificial Intelligence (AI). The AI has been trained on a large dataset and can make recommendations for your decisions. The AI, like all other AIs, is not perfect, there is no guarantee that the AI's recommendations are correct. Note that while you will have access to 12 pieces of information in each round, the AI can only utilize 6.

if Treatment == No Choice:

The 6 pieces of information that the AI utilizes have been pre-selected by

if Method == Algorithm:

an algorithm.

else if Method == Combination:

an algorithm and an expert in the respective domain

else if Method == Expert:

an expert in the respective domain.

end if

else if Treatment == Choice:

How the six pieces of information have been pre-selected depends indirectly on you for each domain. On the page where the domain details are explained, you can choose if the information should be pre-selected by an algorithm, an expert in the respective domain or a combination of both.

end if

If the AI's recommendation differs from your initial decision, you will have the opportunity to reconsider your decision on a new page. Remember, your goal is not to reach a consensus with the AI but rather to make the most correct decisions.

Payment You will receive a fixed payment of £5 for participating in the study. There is a performance-based bonus that depends on the correctness of your decisions. In each round, you can earn an additional £0.20 when your decision is correct. With a total of 40 rounds, the maximum bonus payment is £8. You will not receive immediate feedback about the correctness of your decisions. However, at the end of the study, you will receive an overview of your bonus payments.

Survey Upon completion of all domains and their task rounds, you will be asked to complete a survey. This survey will include questions about your personality, your knowledge of the domains, and your experience with AI systems.

Comprehension Check To ensure that you have thoroughly understood these instructions, you will need to answer a set of comprehension questions. Please be aware that if you fail to answer one out of these questions correctly after three attempts, you will be unable to continue with the study."

S1 Text.
