# Supplementary material for: Algorithm, expert, or both? Evaluating the role of feature selection methods on user preferences and reliance
Source: PLoS One. 2025 Mar 7;20(3):e0318874. doi: 10.1371/journal.pone.0318874 (PMC11888136; doi:10.1371/journal.pone.0318874)
Supplement: S1 Fig — (PDF) [file pone.0318874.s002.pdf]

S1 Figure

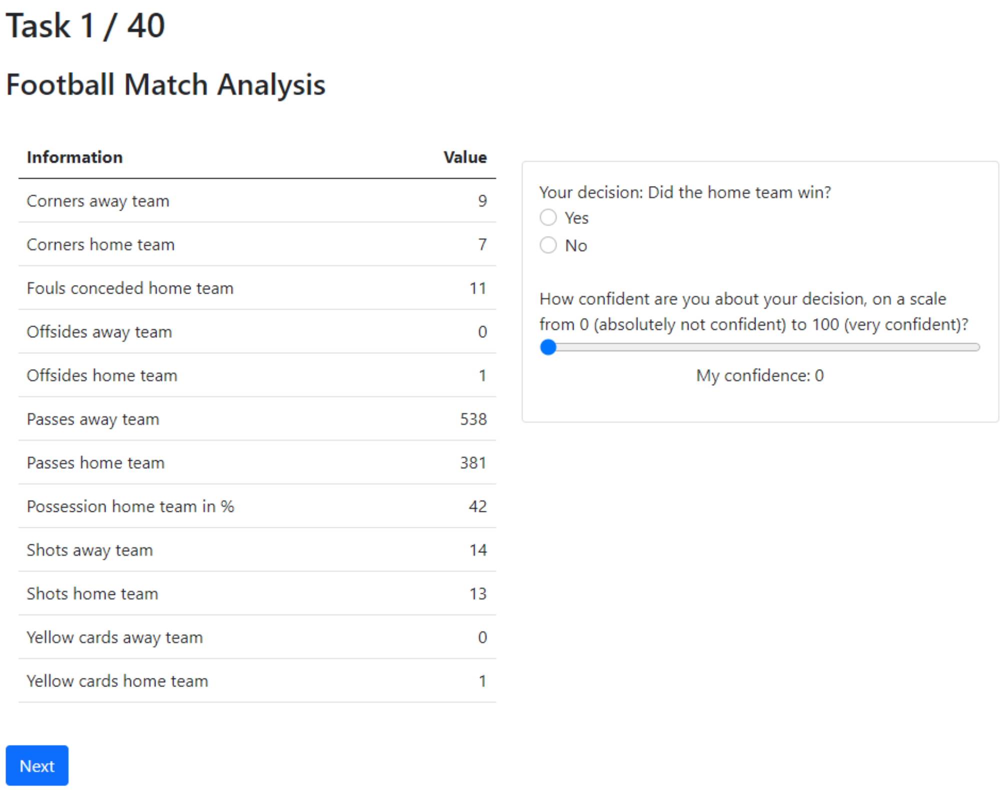

Information	Value
Corners away team	9
Corners home team	7
Fouls conceded home team	11
Offsides away team	0
Offsides home team	1
Passes away team	538
Passes home team	381
Possession home team in %	42
Shots away team	14
Shots home team	13
Yellow cards away team	0
Yellow cards home team	1

S1 Figure. Screenshot of the initial decision page. On the left side, the information for the current decision is displayed. On the right side, participants provide their decision and indicate their confidence level.
