# Supplementary material for: Algorithm, expert, or both? Evaluating the role of feature selection methods on user preferences and reliance
Source: PLoS One. 2025 Mar 7;20(3):e0318874. doi: 10.1371/journal.pone.0318874 (PMC11888136; doi:10.1371/journal.pone.0318874)
Supplement: S2 Fig — (PDF) [file pone.0318874.s003.pdf]

S2 Figure

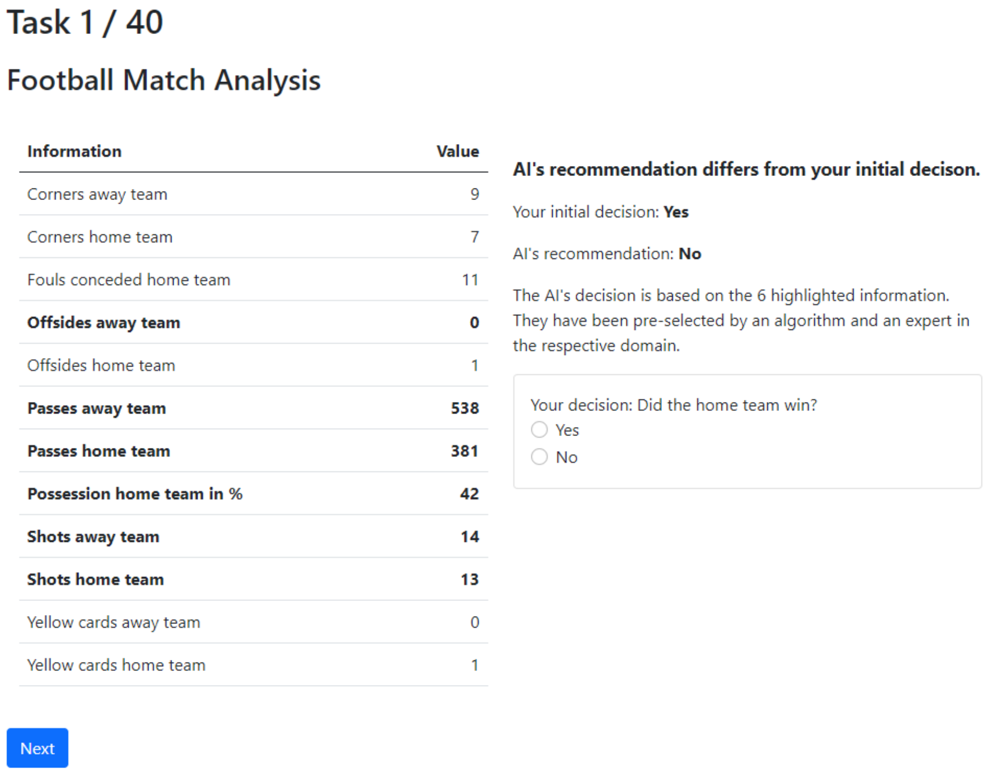

Task 1 / 40

Football Match Analysis

Information	Value
Corners away team	9
Corners home team	7
Fouls conceded home team	11
**Offsides away team**	**0**
Offsides home team	1
**Passes away team**	**538**
**Passes home team**	**381**
**Possession home team in %**	**42**
**Shots away team**	**14**
**Shots home team**	**13**
Yellow cards away team	0
Yellow cards home team	1

AI's recommendation differs from your initial decison.

Your initial decision: **Yes**

AI's recommendation: **No**

The AI's decision is based on the 6 highlighted information. They have been pre-selected by an algorithm and an expert in the respective domain.

Your decision: Did the home team win?
Yes
No

Next

S2 Figure. Screenshot of the subsequent decision page. Similar to the initial decision, participants see the same information on the left side, with the features used by the model now highlighted. On the right side, participants view their previous estimate alongside the model's advice. Below this, they can make their final decision.
