# Supplementary material for: Algorithm, expert, or both? Evaluating the role of feature selection methods on user preferences and reliance
Source: PLoS One. 2025 Mar 7;20(3):e0318874. doi: 10.1371/journal.pone.0318874 (PMC11888136; doi:10.1371/journal.pone.0318874)
Supplement: S1 Data — (PDF) [file pone.0318874.s004.pdf]

S1 Data

Experimental data and analysis scripts can be found at

https://osf.io/z2xpy/?view_only=90607651bed949d29593c4a176d6c96d

Dataset for the Cardio domain:

https://www.kaggle.com/datasets/sulianova/cardiovascular-disease-dataset

Dataset for the Football domain:

https://www.kaggle.com/datasets/pablohfreitas/all-premier-league-matches-20102021

S1 Data.
