# Supplementary material for: Algorithm, expert, or both? Evaluating the role of feature selection methods on user preferences and reliance
Source: PLoS One. 2025 Mar 7;20(3):e0318874. doi: 10.1371/journal.pone.0318874 (PMC11888136; doi:10.1371/journal.pone.0318874)
Supplement: Attachment — Submitted filename: Response to Reviewers.pdf [file pone.0318874.s006.pdf]

UNIVERSITÄT PADERBORN

October 11, 2024

Paderborn University
Phone: (+49) 5251 60-2083
Email: jaroslaw.kornowicz@uni-paderborn.de

Regarding: **Rebuttal Letter to Submission PONE-D-24-30693**

Dear Editor and Reviewers,

We sincerely thank the reviewers for their prompt and detailed feedback on our manuscript. In response, we have carefully revised the manuscript, and this rebuttal letter outlines the key improvements made.

Alongside the revised manuscript, a version with highlighted changes has been included for clarity. New text appears in green, modifications in yellow, and deleted elements are crossed out in red.

*Editor: "Please include this amended Role of Funder statement in your cover letter"*
We have changed this to: "JK and KT would like to acknowledge financial support from he German Research Foundation (DFG) in the Collaborative Research Center TRR 318/1 2021 'Constructing Explainability' (438445824). The authors confirm that the funders had no role in the study's design, data collection, analysis, decision to publish, or manuscript preparation.".

*Editor: "Please include your full ethics statement in the 'Methods' section of your manuscript file."*
In the Ethics Statement within the Methods section, the name of the ethics committee was already listed, and details regarding participant involvement have now been added.

*Editor: "We strongly recommend all authors decide on a data sharing plan before acceptance, as the process can be lengthy and hold up publication timelines."*
Our experimental data and analysis code are publicly available through an OSF repository, with the link provided in the manuscript.

*R1: "Very well written paper, over all good work, highly satisfactory analysis but I think scope of improvement in presented approach as the approach needs refinement and still lacks of novelty, it is application of existing approaches, no significant innovative contribution from authors side, we would like to listen in this regard from authors side."*
We have clarified that the novel aspect of our study lies not only in comparing different types of feature selection methods but also, more importantly, in exploring the varying levels of human integration. Our results indicate that these relationships are more complex than initially expected with humans having a strong tendency to favor human involvement in algorithm construction but do not reveal the same preference when confronted with algorithms. This attitude-behavior gap is new to the debate of Human-AI construction.

*R1: "Use of Non-Expert Participants: The study utilizes lay participants instead of domain experts, limiting the generalizability of the findings to real-world professional settings where expertise is crucial. Resolve this limitation in the revised manuscript."*
In response to Reviewer 2's observation regarding the recruitment of non-experts, we acknowledge this as a limitation. This research serves as an initial step for basic investigation, and future studies should verify our findings in practical situations involving real experts. However, we mentioned in the manuscript that we do not expect major differences as single experts may have biased mental models about the decision

problem and its factors, too. In fact, much of the literature reveals that the same biases are prevelant in layman and experts, see e.g. the following sources: Kynn, M. (2008). The 'heuristics and biases' bias in expert elicitation. Journal of the Royal Statistical Society Series A: Statistics in Society, 171(1), 239-264. Specifically for our domains, we rely on previous research comparing experts and layman finding no big differences in their abilities to forecast outcomes, e.g. Butler, D., Butler, R., & Eakins, J. (2021). Expert performance and crowd wisdom: Evidence from English premier league predictions. European Journal of Operational Research, 288(1), 170-182. Still, the difference may be larger in the domain of medical assessments. However, experts and layman should equally likely be in favor for human oversight.

*R2: "Limited Scope of Feature Selection Methods: The study only considers three feature selection methods (algorithm-based, expert-based, and combined), excluding more advanced techniques like dynamic feature selection or metaheuristic optimization, which could provide a more comprehensive exploration of feature selection strategies. Resolve this limitation in the revised manuscript."*
We agree that many other ML-driven approaches to feature selection are available, but central to our paper is the question whether humans want human oversight in feature selection and whether human oversight matters for reliance. We therefore keep the experiment as close as possible to our reseach question, ignoring the huge variance in ML methods. We chose three feature selection methods comprehensible to participants without technical training, as these methods specifically reflect the varying levels of human integration central to our research question.

*R2: "Stated Preferences vs. Actual Reliance Discrepancy: The study highlights a gap between users' stated preferences and their actual reliance on feature selection methods but does not delve deeply into the cognitive reasons behind this discrepancy."*
The comment on cognitive processes leading to observed discrepancies was insightful. As a result, we briefly addressed two explanatory approaches — dual-process theory and social desirability bias — in the Discussion section. In fact, we believe that the revelation of the attitude-behavior gap in our study is the greatest contribution.

*R2: "Domain-Specific Results: The study shows different results in user reliance between domains (e.g., healthcare vs. sports), but does not thoroughly investigate why domain-specific factors influence these outcomes."*
Potential domain effects have also been discussed. Given the consistent risk due to identical payouts and higher confidence in the medical domain, we suspect that participants' perceptions of AI competencies may differ between the medical and sport-related domains. Still, based on a comparison of two domains, we can only speculate about the potential reasons.

*R2: "Subtle Manipulation of Feature Selection Methods: The distinction between the feature selection methods may not have been clear enough to participants, which could dilute the observed effects on decision-making and reliance."*
We agree that our manipulation might be subtle, and this is noted in the limitations section. We believe that the manipulation during the decision-making phase is inconspicuous but therefore more realistic. Note that we have built in attention checks in the experiment to ensure that participants were paying attention to the written text during the experiment.

*R2: "Lack of Long-Term User Behavior Analysis: The experiment captures short-term interactions with AI and does not assess how user preferences and reliance evolve, missing insights into the long-term dynamics of AI trust."*
We also agree that our study only allows for observing short-term effects and that long-term studies are necessary for true external validity. However, we believe that, as a first step, short-term hypotheses should be tested before moving on to the more challenging task of testing long-term hypotheses. Giving that our research question is, whether users prefer human oversight in feature selection, we do not see how repeated and long-term observations will change the results. Concerning the second research question (will humans rely on AI depending on the mode of feature selection), we agree that human reliance may change over time, but more likely not as a result of feature selection mode but rather based on experience wit the specific AI.

*R2: "No Real-Time Feedback on Decisions: Participants did not receive real-time feedback on their decisions, limiting the understanding of how performance feedback might influence reliance on AI systems."*
Although feedback was intentionally withheld from participants during the task, we recognize that direct feedback exists in many real-life situations. However, providing feedback in this study could have introduced unnecessary noise and confounding effects. For instance, participants may have experienced their first choice to be right (versus wrong), and then very different dynamics would evolve. We, therefore, deliberately choose

not to provide feedback to minimize noise. Still, we agree that with a huge pool of individuals and massive funding, adding feedback and observing long-term effects would be valuable.

*R2: "Absence of Task Complexity Analysis: The study does not investigate how the complexity of tasks influences user reliance on different feature selection methods, potentially overlooking a key factor that impacts decision-making."*
Task complexity is a relevant factor, though it extends beyond the scope of the current experiment. While studies show reliance on AI increases with task complexity, in our experiment, complexity remains relatively uniform between domains and across rounds. This has been accounted for in our regression model. Despite using this as a control variable, the role of task complexity is not the research question.

*R2: "Single Decision-Making Context: The study uses only two decision-making domains (healthcare and sports), limiting the generalizability of the results across a wider range of industries and decision-making environments."*
We acknowledge that including more than two domains could enhance the generalizability of our findings. However, to avoid excessively long experiments, we chose to limit the number of domains. Notably, most studies in our field similarly focus on just one domain and by introducing and comparing two domains, we are already advancing the field. Obviously, a greater range is always good and we encourage future research to validate our results in other domains.

*R2: "Focus on Binary Decision Tasks: The study focuses solely on binary decision tasks, which may not fully capture the complexity of real-world decision-making processes that often involve multiple choices or continuous variables."*
Regarding the binary nature of the tasks, we followed existing literature, as binary decisions are generally easier for participants to understand. That said, if the experiment were to be conducted again, we would likely use probabilistic estimates, as they better capture reliance than simple yes/no answers. Yet, many decision systems advice in binary choice sets (yes or no) rather than in probabilistic decision making situations.

*R1: "These few recent state-of-the-art studies should be discussed where human disease prediction related problems have been thoroughly discussed using feature selection."*
*R2: "Discuss the scope and relevance of the following studies in terms of User Preferences and Reliance in your work."*
We would like to thank you for the suggested literature. We have now incorporated it to strengthen the Related Work section.

Once again, we greatly appreciate the reviewers' valuable feedback and believe that these revisions have significantly strengthened our manuscript.

Sincerely yours,

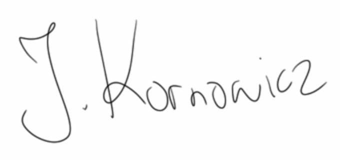


Jaroslaw Kornowicz
