# Supplementary material for: Algorithm, expert, or both? Evaluating the role of feature selection methods on user preferences and reliance
Source: PLoS One. 2025 Mar 7;20(3):e0318874. doi: 10.1371/journal.pone.0318874 (PMC11888136; doi:10.1371/journal.pone.0318874)
Supplement: Attachment — Submitted filename: response_to_reviewers_january.pdf [file pone.0318874.s007.pdf]

UNIVERSITÄT PADERBORN

January 6, 2025

Paderborn University
Phone: (+49) 5251 60-2083
Email: jaroslaw.kornowicz@uni-paderborn.de

Regarding: **Rebuttal Letter to Submission PONE-D-24-30693R1**

Dear Editor and Reviewers,

We would like to once again thank the reviewers for their feedback on our manuscript.

With regard to the following suggestions, we have revised our manuscript. For this, we have highlighted the relevant sections in the manuscript and would like to briefly explain our points in this letter.

Reviewer 3: "1) Please highlight the novelty of the work. 2) What are the limitations of the work?"

Thank you for allowing us to revise these points. We have edited part of the "Introduction" the entire section "Limitations and Implications" as follows, changes are marked:

> *Yet, as far as we know, the question of how feature selection modes contribute to AI reliance has not been systematically analyzed. Nonetheless, feature selection and human preferences for feature selection mechanisms are crucial to understanding a model. The novelty of our study lies in addressing the gap in the literature by examining the effects of different levels of human integration in feature selection on user preferences and reliance.*
>
> *...*
>
> *One potential reason for the missing differences in reliance between the methods might be due to a manipulation that is too subtle. There's a possibility that the methods' signals are too faint within the task to detect an effect corresponding to the significant differences observed in preferences. Despite this, the presentation mirrors real-world scenarios where detailed explanations of AI feature selection methods are rarely provided. Participants were able to review the selected features during the tasks, unlike during the method selection phase. This visibility allowed them to reasonably assess the selection's validity, likely comparing it with their judgment. Consequently, the feature selection method information likely serves as only a minor indicator of the selection's validity, possibly leading to the observed results. Future studies might consider not displaying the features, although this approach could reduce realism.*
>
> *Another limitation impacting the generalizability of our findings is the recruitment of non-professional decision-makers from an online participant pool instead of domain professionals. While we acknowledge that expertise is crucial in many real-world applications, using lay participants offers important advantages, especially in the context of fundamental research like ours. Lay participants provide an opportunity to study baseline human-AI interactions without the influence of pre-existing domain-specific knowledge, allowing us to isolate general behavioral patterns related to trust, preferences, and reliance on AI systems. Future studies could build on this foundation by replicating the experiment with domain experts to enhance the real-world applicability of our findings.*
>
> *Nonetheless, it is plausible that domain experts would not yield substantially different outcomes. On the one hand, the literature reveals that the same biases are prevalent among both laypeople*

> *and experts [93, 94]. On the other hand, a meta-analysis shows that in human-AI collaboration experiments, there are no differences in decision-making performance between professional and non-professional participants [95]. We believe that, in addition to expertise in one's own domain, experience in machine learning and feature selection is also needed to form a strong opinion. With only domain experience, we expect similar results as seen with laymen, both concerning the preference for human oversight and the reliance on AI advice.*
>
> *Another way to expand the research in this study would be to shift the focus from short-term interactions to long-term time horizons, exploring how preferences and reliance evolve over time. Long-term research has often been avoided in the human-AI literature due to its empirical challenges, but previous studies suggest the presence of temporal effects [79].*
>
> *By examining algorithm-based, expert-based, and combined feature selection approaches, we offer fresh insights into how human involvement shapes user trust, preferences, and reliance on AI-driven decisions. Our findings highlight the nuanced and complex relationships between human involvement and user behavior, revealing that the degree of human input can significantly influence perceptions of transparency and trustworthiness, yet these perceptions may not always translate into greater reliance on the system. We reveal a significant attitude-behavior gap, known in many disciplines and for many instances: While humans reveal strong stated preference for human oversight ex ante, individuals are equally likely to rely on AI advice, independent of human oversight.*
>
> *Our results have practical implications, especially when transparency is essential in decision support systems and there is a lack of trust towards them. Those overseeing or designing AI systems could communicate that the data the AI uses was selected from a joint effort between human experts and algorithms. However, they also need to consider individual traits. As AI systems are often developed in this way, making this known might align with users' preferences, potentially increasing the likelihood of using these systems and leading to better decision-making outcomes.*

We hope this revision provides greater clarity and look forward to receiving any further feedback or questions you may have.

Sincerely yours,

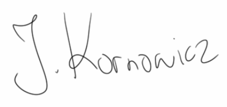

Jaroslaw Kornowicz
