# Supplementary material for: Algorithm, expert, or both? Evaluating the role of feature selection methods on user preferences and reliance
Source: PLoS One. 2025 Mar 7;20(3):e0318874. doi: 10.1371/journal.pone.0318874 (PMC11888136; doi:10.1371/journal.pone.0318874)
Supplement: Attachment — Submitted filename: Revisions.docx [file pone.0318874.s005.docx]

**Paper Title**

Algorithm, Expert, or Both? Evaluating the Role of Feature Selection Methods on User Preferences and Reliance

The paper tackles an important and timely topic in the intersection of **human-computer interaction** and **AI transparency**. As the demand for explainable AI (XAI) grows, understanding how users prefer to engage with feature selection processes is highly relevant. Moreover, the study contributes to the existing literature by addressing the gap in the interaction between **expert-driven** and **algorithmic feature selection**, which has practical implications for AI deployment. Moreover, I have added the following comments to enhance the quality of the work.

**Comments:**

1. **Use of Non-Expert Participants**:
   - The study utilizes lay participants instead of domain experts, limiting the generalizability of the findings to real-world professional settings where expertise is crucial. Resolve this limitation in the revised manuscript.
2. **Limited Scope of Feature Selection Methods**:
   - The study only considers three feature selection methods (algorithm-based, expert-based, and combined), excluding more advanced techniques like dynamic feature selection or metaheuristic optimization, which could provide a more comprehensive exploration of feature selection strategies. Resolve this limitation in the revised manuscript.
3. **Stated Preferences vs. Actual Reliance Discrepancy**:
   - The study highlights a gap between users' stated preferences and their actual reliance on feature selection methods but does not delve deeply into the cognitive reasons behind this discrepancy.
4. **Domain-Specific Results**:
   - The study shows different results in user reliance between domains (e.g., healthcare vs. sports), but does not thoroughly investigate why domain-specific factors influence these outcomes.
5. **Subtle Manipulation of Feature Selection Methods**:
   - The distinction between the feature selection methods may not have been clear enough to participants, which could dilute the observed effects on decision-making and reliance.
6. **Lack of Long-Term User Behavior Analysis**:
   - The experiment captures short-term interactions with AI and does not assess how user preferences and reliance evolve, missing insights into the long-term dynamics of AI trust.
7. **No Real-Time Feedback on Decisions**:
   - Participants did not receive real-time feedback on their decisions, limiting the understanding of how performance feedback might influence reliance on AI systems.
8. **Absence of Task Complexity Analysis**:
   - The study does not investigate how the complexity of tasks influences user reliance on different feature selection methods, potentially overlooking a key factor that impacts decision-making.
9. **Single Decision-Making Context**:
   - The study uses only two decision-making domains (healthcare and sports), limiting the generalizability of the results across a wider range of industries and decision-making environments.
10. **Focus on Binary Decision Tasks**:

- The study focuses solely on binary decision tasks, which may not fully capture the complexity of real-world decision-making processes that often involve multiple choices or continuous variables.

11. **Discuss the scope and relevance of the following studies in terms of User Preferences and Reliance in your work.**

- Tiwari, A., & Chaturvedi, A. (2022). A hybrid feature selection approach based on information theory and dynamic butterfly optimization algorithm for data classification. *Expert Systems with Applications*, *196*, 116621.
- Tiwari, A., & Chaturvedi, A. (2021). A novel channel selection method for BCI classification using dynamic channel relevance. *IEEE Access*, *9*, 126698-126716.
- Tiwari, A., & Chaturvedi, A. (2023). Automatic EEG channel selection for multiclass brain-computer interface classification using multiobjective improved firefly algorithm. *Multimedia Tools and Applications*, *82*(4), 5405-5433.
- Tiwari, A. (2023). A logistic binary Jaya optimization-based channel selection scheme for motor-imagery classification in brain-computer interface. *Expert Systems with Applications*, *223*, 119921.
- Tiwari, A. (2023). A hybrid feature selection method using an improved binary butterfly optimization algorithm and adaptive β–hill climbing. *IEEE Access*, *11*, 93511-93537.
- Yin, L., Wang, L., Lu, S., Wang, R., Ren, H., AlSanad, A.,... Zheng, W. (2024). AFBNet: A Lightweight Adaptive Feature Fusion Module for Super-Resolution Algorithms. Computer Modeling in Engineering & Sciences , 140(3), 2315-2347. doi: https://doi.org/10.32604/cmes.2024.050853
